# Laser Engineering Nanocarbon Phases within Diamond for Science and Electronics

**DOI:** 10.1021/acsnano.3c07116

**Published:** 2024-01-17

**Authors:** Patrick S. Salter, M. Pilar Villar, Fernando Lloret, Daniel F. Reyes, Marta Krueger, Calum S. Henderson, Daniel Araujo, Richard B. Jackman

**Affiliations:** † Department of Engineering Science, University of Oxford, Parks Road, Oxford, OX1 3PJ, U.K.; ‡ Department of the Science of Materials, University of Cadiz, 11510, Puerto Real, Spain; § London Centre for Nanotechnology and Department of Electronic and Electrical Engineering, UCL (University College London), 17−19 Gordon Street, London, WC1H 0AH, U.K.

**Keywords:** diamond, laser processing, electronic devices, graphitic wires, meteorites

## Abstract

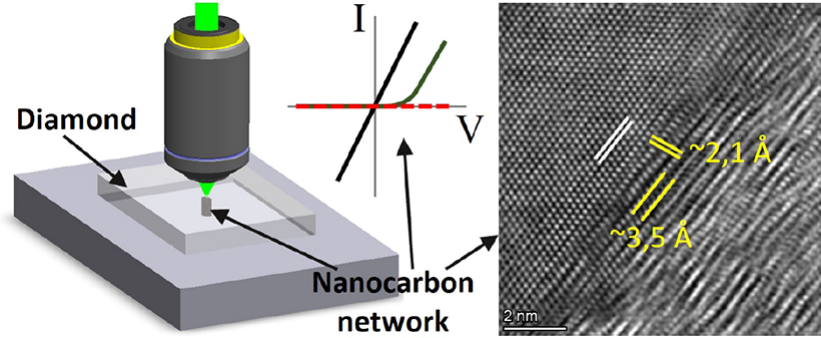

Diamond, as the densest allotrope of carbon, displays a range of exemplary material properties that are attractive from a device perspective. Despite diamond displaying high carbon–carbon bond strength, ultrashort (femtosecond) pulse laser radiation can provide sufficient energy for highly localized internal breakdown of the diamond lattice. The less-dense carbon structures generated on lattice breakdown are subject to significant pressure from the surrounding diamond matrix, leading to highly unusual formation conditions. By tailoring the laser dose delivered to the diamond, it is shown that it is possible to create continuously modified internal tracks with varying electrical conduction properties. In addition to the widely reported conducting tracks, conditions leading to semiconducting and insulating written tracks have been identified. High-resolution transmission electron microscopy (HRTEM) is used to visualize the structural transformations taking place and provide insight into the different conduction regimes. The HRTEM reveals a highly diverse range of nanocarbon structures are generated by the laser irradiation, including many signatures for different so-called diaphite complexes, which have been seen in meteorite samples and seem to mediate the laser-induced breakdown of the diamond. This work offers insight into possible formation methods for the diamond and related nanocarbon phases found in meteorites.

## Introduction

Carbon is one of the most abundant elements on Earth and forms a rich assortment of allotropes, with well-known examples of diamond and graphite in addition to more exotic structures such as graphene, fullerene, and carbon nanotubes. A common factor linking the various allotropes is their importance for both technological applications and scientific discovery. This is exemplified by diamond, where natural stones are an important geological resource,^1^ while recent advances in laboratory-based diamond growth have driven forward applications where the properties of diamond as a wide band gap semiconductor with extreme electronic, optical, and thermal properties can be exploited.^2^ In addition to electronics,^3^ electrochemical devices^4,5^ and biomedical devices^6,7^ fabricated from laboratory-grown diamond have become of significant interest. Recently, the stable nature of the nitrogen vacancy (NV^*–*^) and other defect centers within diamond has generated great interest in diamond as a platform for quantum technology and its many applications.^8^

Traces of diamond have even been discovered in meteorites, with the suggestion that these crystals are formed as a metastable phase in stellar condensation long before they reach Earth.^9^ These diamonds are typically 2–3 nm clusters consisting of a diamond core surrounded by a fullerene-like network,^10^ drawing comparison with laboratory-made “detonation nanodiamonds”.^11^ In contrast, other reports argue that the diamonds are formed within the meteorites upon Earth impact, typically being larger in mostly two- or three-phase aggregates (diamond, lonsdaleite, graphite);^12^ in such cases they were considered to show morphological and microstructural features related to a solid-phase transition of graphite to diamond from the shock impact. However, recently when meteorite “impact” diamonds have been compared with those synthesized by lab-based detonation experiments, distinct differences were apparent,^13^ such that complete analogues of natural impact diamonds are yet to have been synthesized artificially. Despite the importance within the field of planetary science, the origin of nm−μm diamond in meteorites, particularly the carbon-rich ureilites, is still a subject of some debate.^14^ In this work, laser-based laboratory methods are used to engineer relevant complex nm−μm-scale diamond features and other exotic carbon phases, as well as indicate how these can be tailored for effective use in modern technology, such as electronics.

High-quality single-crystal diamond substrates, produced by chemical vapor deposition methods, are now readily grown in the laboratory and are commercially available.^15^ As diamond is the hardest known natural material, it can be challenging to effectively process the raw diamond substrate to manufacture devices. Over the past decade, laser processing with sub-picosecond duration pulses has become established as a useful technique for internal modification of so-called lab-grown diamond.^16^ Such laser processing has largely focused on the fabrication of electrically conductive vias that pass through a diamond wafer with device applications mainly focused on radiation detectors.^17,18^ Recent advances in adaptive optical control over the laser focus^19^ have enabled an advanced generation of laser-written diamond devices for quantum technology to be developed.^20,21^ Indeed, we show here that such control combined with specific processing conditions can also give rise to electrical properties of laser-written tracks that have not been previously reported.

Initially the laser-written tracks in diamond were broadly defined as “graphitic” based upon data from Raman spectroscopy,^16,22^ and efforts were made to engineer the tracks to have as high an electrical conduction as possible.^23^ However, studies with low-resolution transmission electron microscopy (TEM)^24,25^ and scanning electron microscopy (SEM)^26^ have indicated that the internal structure might be more complex, while more recent reports have suggested a complicated relationship between the delivered laser dose and ohmic conductivity.^27^

In this work, high-resolution TEM/STEM (HRTEM/HRSTEM) is used to reveal a surprisingly rich assortment of nanocarbon networks (NCNs) that are generated by the laser write process in diamond, with different network elements influenced by the laser dose (number of laser pulses delivered over a specified time) and the laser process history. We clearly identify a range of structures containing composite sp^3^- and sp^2^-bonding patterns, which appear closely related to the variety of nanocomposite carbon structures found in meteoritic diamond, including the so-called “diaphite” phase(s).^28,29^ We are thus able to replicate the extreme pressure and temperature conditions thought to be needed for formation of these exotic carbon nanostructures,^30^ from an existing diamond basis. This is achieved by accurately delivering focused laser energy into a micrometer scale volume in a femtosecond time frame, with the surrounding diamond matrix providing a high-pressure environment. We also show from a technological perspective that by laser engineering the diamond in this manner, the different electrical conduction mechanisms in the NCNs can be controlled; previous work has shown the formation of conductive tracks from a fs-laser processing; here both junction-like (semiconducting) and insulating tracks are also demonstrated. This holds great promise for future devices, where NCNs can be controllably manufactured within a diamond wafer, providing not only buried conductive wires but 3D structures with active device junctions (depletion or tunneling).

## Results and Discussion

**Figure 1:**
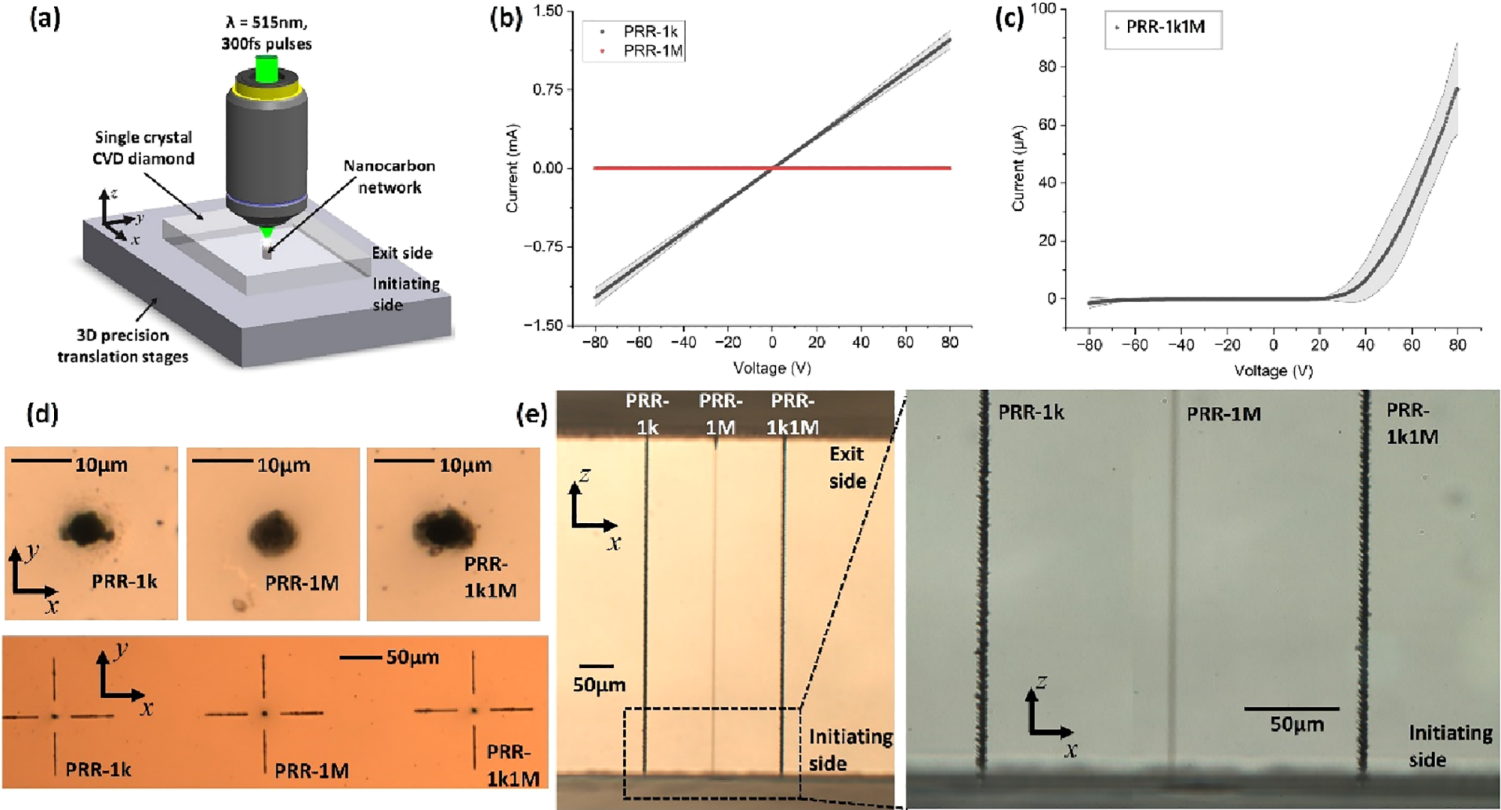
(a) Schematic of the laser processing of NCN columns through a single-crystal diamond wafer. (b) Different dc conduction for NCNs dependent on the laser pulse repetition rate used for fabrication, PRR-1k and PRR-1M, showing stark differences, with ohmic conduction for PRR-1k while insulating characteristics for PRR-1 M conditions. (c) The nonlinear diode-like behavior that can be accessed using a hybrid laser write strategy, where the NCN is initially formed with a PRR of 1 kHz and then overwritten at 1 MHz (PRR-1k1M). (d) Optical micrographs showing the initiating side for each NCN column, including laser-written surface cross-hairs in the lower image for accurate extraction of a lamella through the NCN for TEM inspection. (e) Optical micrographs showing the cross-sectional images of the NCN “wires” from the initiating write-side through the thickness of the diamond plate.

The nanocarbon networks were laser machined inside high-purity single-crystal diamond, as shown schematically in Figure 1(a), with full details in the online methods. The laser was focused down to a spot size of 1 μm on the rear side of the diamond wafer (“initiating side”) to initially form the NCN, with the substrate then translated downward along the optical axis (corresponding to the [100] direction in the crystal) to form a complete NCN column through the wafer. Upon electrical analysis of the fabricated NCNs, three laser processing regimes were identified as showing distinct electrical conduction characteristics, as seen in Figure 1(b) and (c). The processing regimes were dictated by the laser pulse repetition rate (PRR), which when set to 1 kHz yielded NCNs with ohmic conduction, while if the PRR was increased to 1 MHz, the NCN became insulating (Figure 1(b)). Additionally, a hybrid configuration could be accessed by initially forming the NCN column with a single pass of the laser at 1 kHz PRR and then overwriting at 1 MHz, resulting in NCNs with semiconducting properties and showing diode-like behavior (Figure 1(c)). The laser pulse energy of 120 nJ and sample translation speed of 10 μm/s were constant throughout, so it becomes apparent that the PRR is critical in determining the electrical characteristics of the written NCN wires. The three conditions are referred to as PRR-1k, PRR-1M, and PRR-1k1M, respectively. It is important to note that the laser process was initiated at the rear of the sample with the laser beam being subsequently moved upward toward the top of the wafer. As such, the energy deposition profile may differ slightly from the initiation side, where pure diamond is encountered by the incoming photons, to the top side where the laser photons are progressively moving from modified to nonmodified material as they break the top surface. In the case of the results presented here, TEM imaging has been carried out on the “initiating” side of the written lines.

Laser-written structures displaying ohmic electrical conductivity formed from sub-picosecond pulses at a repetition rate (PRR) of 1 kHz have been observed for several years.^31^ Such ohmic “wires” within diamond have been used widely for the fabrication of a range of devices and in particular for 3D detector structures.^32^ It is only very recently that investigations of processing at different PRR have been reported, with observations that increasing the laser dose (number of laser pulses interacting with each volume of material, which is equivalent to increasing the PRR at fixed translation speed) impedes the electrical conduction of the NCN.^27^ It has also been shown that a barrier potential can exist for the NCN when laser parameters were not optimized, but the barrier was symmetric with regard to voltage polarity.^33^ Here we show that through a combination of two differing PRRs applied in sequence, conditions can be created that lead to an asymmetric potential barrier, i.e., conduction in one direction only at a given threshold voltage (Figure 1(c)). It is demonstrated that this unexpected but technologically exciting phenomenon can arise due to the distinct nature of the interacting NCNs formed in different laser processing regimes. Diode-like behavior lies at the heart of most active electronic devices, offering both signal rectification and, in the context of the channel region of a transistor with a gate, the means for channel current modulation that gives rise to the transferred-resistance (transistor) action.

To understand the mechanism for the different conduction properties of the NCNs and how they depend on the laser write conditions, high-resolution imaging of the NCN structure is vital. Figure 1(d) reveals images from a transmission optical microscope of NCN at the initiating side for each of the three laser write conditions. Even though the columns show vastly different electrical conduction properties, they appear surprisingly similar in these optical images with a column diameter of 5–6 μm, showing dark contrast against the bright background from the surrounding diamond. It should be recalled that the focused laser spot employed, being *∼*1 μm in diameter, is significantly smaller than the dimensions of the dark, laser-induced modifications in these optical images. In Figure 1(e) when the laser-written columns are viewed from the side, differences in the structural transformation start to emerge. It is clear that the NCN “wire” written under the condition PRR-1M is optically distinct, being lighter in color and smoother than the NCN “wires” for the PRR-1k and PRR-1k1M. However, these latter two conditions still seem indistinguishable optically, and it is evident that imaging at higher spatial resolution is necessary to identify the nanoscale structural features driving the different electrical characteristics. Identical devices as used in the electrical analysis were therefore prepared for high-resolution transmission electron microscopy (HRTEM/HRSTEM). Electron-transparent lamellae were made using a focused ion beam (FIB) by a lift-out method;^34^ the lamellae were extracted from the center of each laser-induced structure, which were easily observable by SEM.

**Figure 2:**
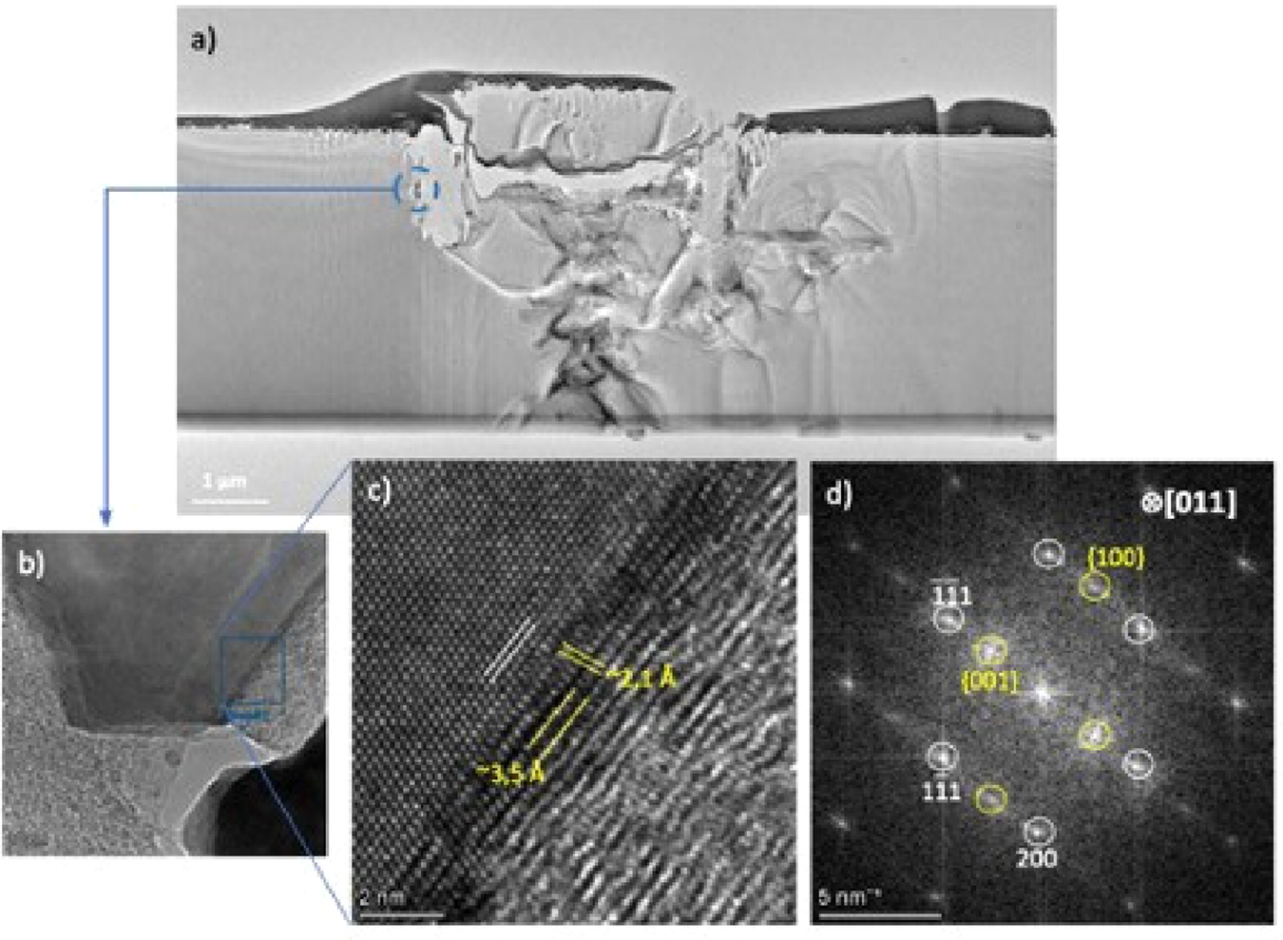
(a) TEM micrograph of diamond FIB lamella containing an NCN displaying ohmic conduction, displaying a “backbone”-type structure throughout the laser-processed column. The area contained in the blue circle is shown in higher resolution in (b), as an example of microstructure within the NCN column. This region is representative of the structures seen lower in the laser-processed area (any of the “darker” regions within the backbone), although the images were lower resolution due to increased thickness of the lamella lower down due to sample preparation issues (Figure S1). (c) HRTEM (unfiltered) micrograph corresponding to the blue square in (b), where a transition from diamond to graphite is shown. White lines mark 111-type diamond planes with spacings of ∼2.1 Å, and yellow lines mark both 100- and 001-type graphite-like (graphene) planes with spacings of ∼2.1 and ∼3.5 Å, respectively. This interfacial region resembles a so-called type 1 “diaphite” phase.^28^ (d) FFT of the full HRTEM image in (c), showing diamond reflections (white circles) corresponding to *B* = [011] and confirming the 100 and 001 reflections (yellow circles) for graphite from the planes described in (c).

Figure 2(a) reveals the internal microstructure of a typical NCN column fabricated with a laser pulse repetition rate of 1 kHz (PRR-1k), where large-scale disruption of the diamond lattice can be seen on the path traced by the laser focus. The light– matter interaction produces a clear backbone structure, of dimension approximately 1 μm across, which propagates through the extent of the lamella and is expected to continue in this manner through the thickness of the diamond wafer. Close to the initiating surface at which the laser beam initially interacts with the diamond, there is some partial ejection of material in an area greater than the laser beam diameter (the dark material on the top of the image can be disregarded, as it is metal associated with the FIB preparation method).

The laser-written column contains characteristic regions of strong lattice damage propagating short distances in [110] directions, as seen in previous studies.^24,26^ These can be understood as the lattice succumbing to accumulated local pressure along the weaker (111) cleavage planes, which intersect with the (110) face of the FIB lamella. When a stationary laser spot is focused beneath the diamond surface, a wave of opaque material is reported to spontaneously propagate through the diamond toward the irradiating laser.^16^ The opaque material was characterized by Raman microscopy to exhibit a “G” peak (*∼*1580 cm^*–*1^), which is typical of graphite.^35^ Under uniform motion of the laser focus through the bulk crystal toward the radiation source, the graphitization wave can propagate in an unlimited manner, enabling embedded graphitic wires to be formed of an arbitrary length.^36,37^ The wires are often characterized as a series of unique wave fronts propagating forward with seemingly sufficient overlap to allow electrical conductivity to occur. The “wavefront” pattern arises as the volume expansion required when forming graphitic material from diamond is “quenched” by the buildup of pressure until the laser spot has moved sufficiently that a less pressured crystal region is encountered. In the previously reported cases the wave fronts are characteristic of the backbone structure observed here with condition “PRR-1k”, as illustrated in Figure 2(a).

In the current study, the use of higher resolution electron microscopy has allowed insight into the structure of these features at an atomic level. The highlighted region in Figure 2(a) is further magnified in Figure 2(b) and is representative of several regions investigated throughout the “backbone” structure created by the laser. While the affected regions are surrounded by sp^3^ crystalline diamond, there are clear sp^2^ graphitic paths running through the processed material, as indicated in the lower right section of Figure 2(c). Of considerable interest is the existence of a transitionary phase between sp^2^- and crystalline sp^3^-bonded carbon. Within the image the white lines mark 111-type diamond planes with a spacing of *∼*2.1 Å, and yellow lines mark both 100- and 001-type graphite planes with spacings of *∼*2.1 and *∼*3.5 Å. This type of phase has been observed in localized regions in femtosecond laser experiments aimed at the generation of diamond from graphitic material.^38^ The diamond-graphene crystalline nanostructure termed “diaphite” has also been predicted by theory following photoexcitation of crystalline sp^2^ carbon.^39^

Intriguingly, diaphite forms have also been found localized between adjoining diamond phases in shock-impact meteorite materials.^29^ In these instances, diaphite was discussed as a nanocomposite material important for structural stability. Despite its high compressive and tensile strength, diamond exhibits a low fracture resistance as a result of crack propagation along the cleavage planes. Inclusion of the diaphite nanocomposite structures may allow the material to absorb or deflect incipient crack formation, thus increasing its fracture resistance.^40^ Here, we suggest that the diaphite contributes a similar functionality in the mediation of the diamond lattice breakdown during laser processing to maintain the structural integrity of the diamond wafer. The demonstration that elongated, near-continuous regions of diaphite, type 1 diaphite in the terminology proposed by Nemeth and co-workers,^29^ can be engineered by this form of laser processing is exciting in both technological and scientific terms, as discussed further below. It is of interest to note that the kHz laser conditions used in this work will deposit sufficient energy at the center of the laser spot to generate the so-called diaphite type 1 phase. The calculations offered by Nemeth and colleagues reveal that this phase is the most energetically expensive form of nanocarbon phase of all those explored, with regard to meteoric nanocarbons.^28^ This suggests that we have been able to replicate in the laboratory the conditions relevant to the formation of the complex nanocarbon phases, including nanodiamonds, found in meteorites.

**Figure 3:**
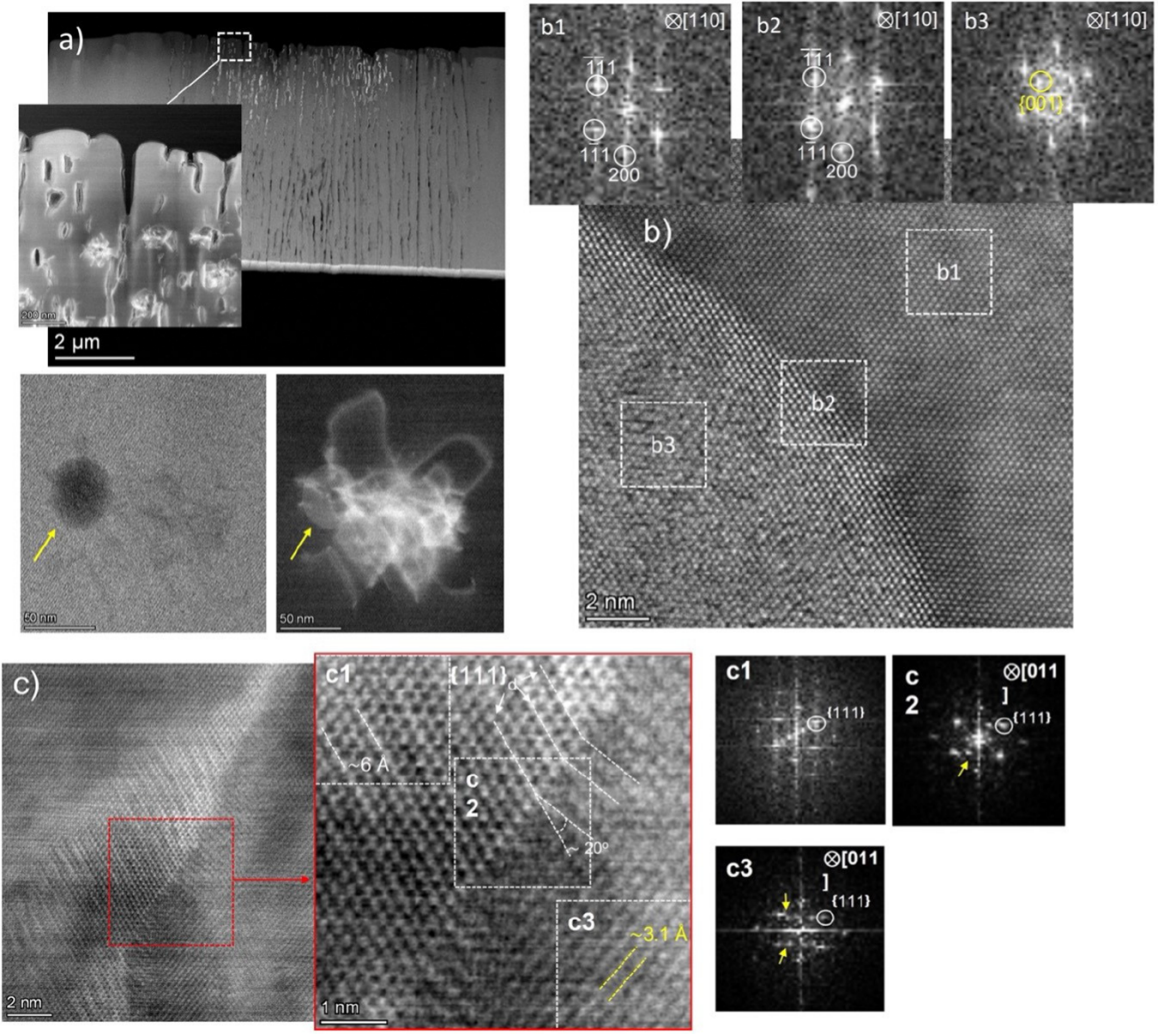
NCN produced by a laser at a high pulse repetition rate, PRR-1M. (a) Top: General TEM image of diamond FIB lamella reveals a completely different structural modification when the laser pulse repetition is increased by a factor of 1000. A series of discrete microscale “holes” with high aspect ratio form across a 6 μm diameter of the column, considerably larger than the ∼1 μm diameter of the beam. The inset shows a dark field image (STEM mode) of the region in the dashed white square, where the formation of densely tangled groups of dislocations surrounding many of these holes is evidenced. Bottom: BF (left) and DF (right) images of one of these “holes” in detail (indicated with the arrow). DF is necessary to reveal these structural defects induced in the diamond by the laser pulse repetition. (b) Nonfiltered HR-STEM micrographs of the interface at the edge of a typical “hole”, with adjacent FFTs of the labeled regions. (c) Nonfiltered HR-STEM micrograph of additional features arising from the laser interaction. An interface region (highlighted by the red squares) is shown in greater detail. FFTs of regions labeled as c2 and c3 indicate the appearance of new reflections consistent with 111 planes of orthorhombic graphite. Evidence of transformation of 111 planes of diamond into 112 or 220 planes of orthorhombic graphite is also observed, as highlighted with dashed lines along the interface. Only partial transformation is realized since diamond reflections are also present in all FFTs.

The nanostructure of the engineered NCNs produced here is strongly dependent on the PRR of the writing laser, as can be seen in Figure 3 when the PRR is increased by 3 orders of magnitude relative to the structures introduced in Figure 2. Indeed, the low-magnification TEM image in Figure 3(a) shows a completely different structural modification. The form of the modification is now much more uniform across a broad diameter of the column, extending to *∼*6 μm. The focused laser spot is of dimensions 1 μm across, so the structural modification extends far beyond the region of maximum laser intensity. In the upper part of Figure 3(a) high-aspect-ratio microscale “channels” appear to be induced over the 6 μm diameter of modified material, extending continuously over several microns or more. However, at higher magnification (Figure 3(a) inset), it is apparent that these channels are not interconnected, and a series of smaller possible voids or holes are also visible in the modified region. The lower part of Figure 3(a) provides greater detail of one such structure, with both bright field (BF) and dark field (DF) images, providing some indication of the formation mechanism. In the BF image, a small, isolated “void” some 30 nm across is visible. The same region imaged in DF reveals a dense collection of tangled dislocations surrounding the void. Nonfiltered HRSTEM micrographs in Figure 3(b) provide further detail of the interface region around the edge of this void. The void does not pass completely through the lamella, and fast Fourier transforms (FFTs) of the structure show high-quality single-crystal diamond both inside and surrounding the void. The contrast in the BF image (marked as positions 1, 2, and 3) thus derives from different material thickness in these regions, indicating that an approximately spherical void has been induced in the diamond by the laser process. In the case of positions 1 and 2, this is confirmed by the FFTs displayed alongside the BF HRTEM image, where spot assignments confirm high-quality crystalline sp^3^-bonded carbon. However, the FFT for region 3, within the void region, reveals an additional ring-like structure with 2 spots aligned with the diamond (111), corresponding to a spacing of 3.7 Å. It would appear reasonable to surmise that during sample preparation any “softer” sp^2^ phases present in the void will have been etched away, leaving a thin more tenacious nondiamond phase attached to the edge of the void. In this case, the presence of additional point-like spots, and not arc-like features, indicates a residue of a “diaphite”-type phase is present in the micrograph at position 3, with a layer spacing of some 3.1 Å due to the “missing” neighboring 3D-graphite that has been removed. The dense collection of dislocations that propagate seemingly randomly from and around all such features (Figure 3(a)) is further evidence that the crystal experienced significant strain when (expansive) sp^2^ material formed in these regions.

It is interesting to note that birefringence images for the diamond following laser processing show a lack of localized crystalline stress for PRR-1M as compared to the apparent high stress around the laser-written columns following PRR-1k (Figure S2); the high density and extensive distribution of dislocation defects apparent in Figure 3 likely occur to reduce otherwise catastrophic levels of stress within the crystal for condition PRR-1M. In other regions spatially separated from the highly damaged and dislocated locations, additional laser-driven phase transformations are observed. Figure 3(c) shows just such a region where a distinct change in the crystallographic arrangement appears at a junction running from the bottom left of the image to the upper right. A magnified portion of this micrograph, indicated by the dotted red box, is also shown in Figure 3(c) along with FFTs recorded at the labeled positions. FFTs 1, 2, and 3 indicate the appearance of some reflections not seen in the region illustrated in Figure 3(b), which are consistent with 111 planes of orthorhombic graphite. Some evidence of transformation of 111 planes of diamond into 112 or 220 planes of orthorhombic graphite is also indicated here as the dashed lines along the interface. Only partial transformation is observed, since diamond reflections are also present. This region shows similarities to the so-called “type 2” diaphite nanocarbon composites reported within meteorite materials by Nemeth and co-workers.^28^ While this laser condition deposits energy at a rate 1000 times higher than the PRR-1k case, the intuitive idea that the resulting structures should therefore have more electrical conductivity but in fact become highly insulating instead can now be understood. The damage created within the diamond crystal is significant and highly localized, with the creation of an extensive network of dislocation threads to reduce crystal stress. No continuous track for the electrical conduction emerges.

**Figure 4:**
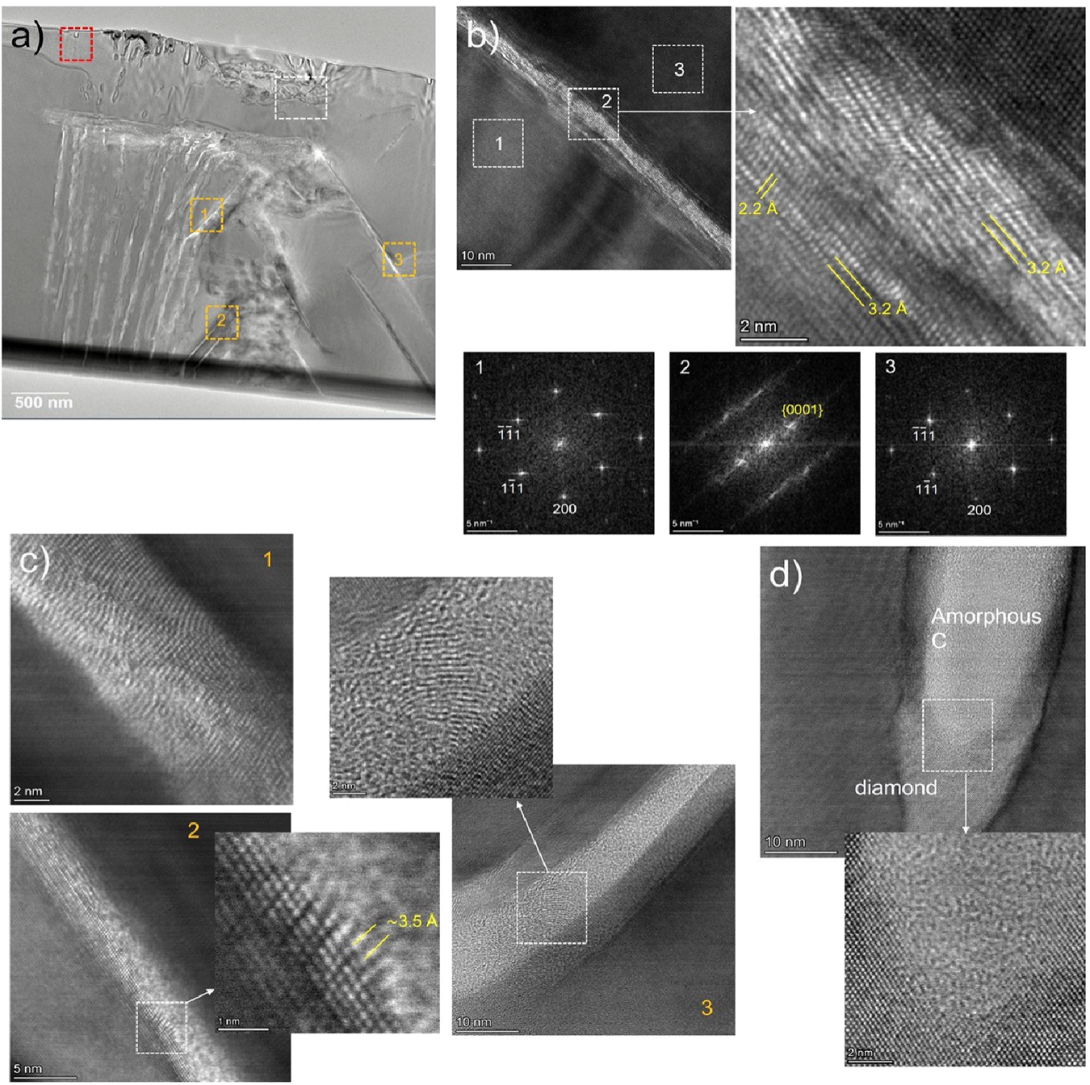
Effects of laser overwriting on a single-crystal diamond. (a) Overview of sample PRR-1k1M. The backbone structure from the precursor PRR-1k laser fabrication can still be seen but is significantly modified by laser overwriting at PRR-1M. (b) Nonfiltered HRTEM micrograph of the region marked with the white dashed square in (a), corresponding to a laser-induced modification in which several layers of highly oriented graphite are inserted between diamond. Three representative areas have been labeled from 1 to 3. Nonfiltered HRTEM micrograph from cropped region labeled as 2 is shown at the right, and the corresponding FFTs 1–3 are shown at the bottom. At the interface, 111 diamond planes become 0001 graphite ones, bearing resemblance to a type 1 diaphite structure. Planar spacings for 0001 and 1011 planes of graphite (∼3.2 and ∼2.2 Å, respectively) are also shown. However, further in to the “backbone” structure, the mediating diaphite feature is not observed and instead graphite or amorphous carbon appears to form a direct interface with the diamond. (c) Three BF HR-STEM nonfiltered micrographs showing examples of amorphous carbon and/or graphite in regions marked with orange squares in (a). (d) Amorphous carbon/diamond interface in the small hole in the red square in (a).

As the two conditions, PRR-1k and PRR-1M, give rise to such divergent outcomes, it is of interest to consider the outcome of sequential laser treatments. The chosen conditions were a first laser pass as per PRR-1k followed by a second pass with the conditions of PRR-1M; in both cases, the laser was drawn from the rear (initiating) side of the diamond up toward the top surface. During the second pass of the laser, the existing structure from the first pass causes an amplitude aberration of the laser beam, which varies as a function of depth. However, focusing with 0.5 NA the aberration is minimal at the rear initiating side from where the lamellae are extracted for TEM analysis. Figure 4 reveals the laser-induced structural modifications for the overwritten structure, known as PRR-1k1M, which displayed unusual electrical conductivity with an asymmetric barrier potential as observed in Figure 1(c). The conductive backbone structure from the PRR-1k precursor can be clearly identified, but the overwrite of PRR-1 M has caused significant structural alteration compared with the situation in Figure 2(a). Figure 4(a) shows an overview of the affected region. In contrast to the PRR-1k precursor, the overwrite appears to have removed a significant proportion of the (darker) sp^2^-bonded graphite content in the “backbone”. Although the backbone structure is still visible, it seems to be dominated by features consistent with polycrystalline diamond. It is not just the backbone structure of the PRR-1k1M features that shows different microstructure from the PRR-1k conductive precursor. As found in the case of PRR-1M, the PRR-1k1M process-modified material extends considerably beyond the *∼*1 μm width of the laser spot size. There are noticeable linear cracks arising from the backbone and extending several hundred nanometers into the diamond bulk along the (111) direction. Additionally, no graphite is observed in the surface region, only diaphite–diamond interfaces. However, at distances beyond 1 μm deeper in the backbone structure, only graphite–diamond interfaces are observed (Figure 4). In Figure 4(b), high-resolution images of the region, indicated by the white box in (a), show the diaphite–diamond interfaces, whereas in Figure 4(c) and (d), only graphite or amorphous-carbon interfaces to diamond can be observed. In the case of (b), three representative areas have been labeled from 1 to 3, shown as both HRTEMs and corresponding FFTs. While the regions labeled as 1 and 3 correspond to diamond, at the interface, labeled 2, 111 diamond planes become 0001 graphite ones, bearing resemblance to a type 1 diaphite structure.^28^ Planar spacings for 0001 and 1011 planes of graphite (*∼*3.2 and *∼*2.2 Å, respectively) are also shown.

Figure 4(c) shows higher magnification images for the three orange squares indicated in Figure 4(a). Here it can be seen that the “cracks” are actually full of either graphite or amorphous carbon, dependent on the size of the sampled crack. In such features of the PRR-1k precursor, the basal planes of graphite are typically aligned parallel with respect to the crystal (as in type 1 diaphite, where graphene-like planes form at an interface). In contrast, here in the PRR-1k1M structure, all of the graphite observed was oriented differently, either transverse to the feature direction or in large, curved configurations as seen in Figure 4(c). Furthermore, while we are able to again identify type 1 diaphite structures within 500 nm from the air–diamond interface (Figure 4(b)), these interphase regions disappear moving deeper into the diamond. For the deeper cracks (Figure 4(c)), there is no clear interphase region, and there appears a direct transition from diamond to graphite, which is again in contrast to the PRR-1k precursor. In terms of electrical conduction, that this treatment results in asymmetry in the *I*–*V* curve (Figure 1(c)) is both intriguing and technologically exciting, giving rise to the concept of active electronic structures embedded within diamond.

The results presented above enable the electrical properties of these laser-written “wires” shown in Figure 1(b,c) to be understood. For wires created using PRR-1k conditions, the *I*–*V* characteristics are ohmic in that electrical conduction occurs in both positive and negative charge directions with the same characteristic resistance. The TEM images presented here (Figure 2) show that far from the creation of a single cohesive “wire” an extensive array of complex NCNs is created. That these networks are sufficiently extensive to be interconnected and contain both graphite-like and graphene-like regions explains the ohmic conductivity observed. Although the “backbone” structure seen (Figure 2(a)) appears to have repeating periodicity as the laser spot moves through the diamond, the NCNs are clearly sufficiently connected as to allow barrier-free electrical conduction. Although intuitively increasing the laser pulse rate by 1000 times may be considered to reduce the resistance of the wire through the creation of more extensive NCNs, the contrary is the case in that conductivity is quenched, and the wire, though still optically visible, becomes highly electrically insulating. This can be understood in terms of the pressure that builds up within the crystal as sp^3^ material is transformed to the less dense form of carbon, sp^2^. Given the energy from the laser is delivered in the form of femtosecond pulses, little thermal dissipation can occur since bond vibrations tend to be measured in tens of picoseconds.^41^ Thus, as the laser spot moves with the same velocity in both cases, under the conditions of PRR-1M pulses will “build” up the energy confined in the system with the more catastrophic forms of damage observed in the TEM images. Indeed, in Figure 3(a) significant damage in the form of cracks and voids can be seen near the surface region, where material ejection may be possible. Deeper in the affected region where such material release is no longer possible the damage reveals itself as extensive networks of thread dislocations and sp^3^ “voids” containing isolated graphitic-like material. This type of gross but localized damage to the crystal shows insufficient connectivity between conductive regions to allow any form of electrical conduction.

Near the surface of the diamond, NCNs similar to those seen in Figure 2 for PRR-1k can be seen, presumably because of surface expansion. Once the surface has been left (*∼*500 nm), the crystal instead relaxes through the propagation of extensive networks of dislocation threads in all directions. No interconnected sp^2^-like regions exist, and conductivity is lost.

The concept of electrical percolation within composite systems is well established;^42^ indeed the recent application of complex network theory to electrical conduction in nanostructure assemblies allows for a wide range of conduction types to be observed in assemblies that appear at first sight to be discontinuous.^43^ In essence, at a certain concentration discontinuous conductive regions will be sufficiently close and numerous as to allow either direct, but random, connectivity or tunneling at a given threshold field and hence the onset of conduction. Such processes usually result in symmetric conductivity, all with an onset threshold voltage. However, there are reported circumstances where nanocarbon systems display asymmetric current flow when a field is applied. Carbon nanotubes (CNTs) with differing chirality can be excellent metals or semiconductors with a band gap that is inversely proportional to their diameter.^44^ Perhaps of more significance here is the observation that graphene nanoribbons (GNRs), that is, strips of graphene 10 nm or less in width, become semiconductors.^45,46^ The graphene planes associated with the so-called diaphite phase meet the theoretical criteria for this form of electronic behavior.^47^ According to the results presented in Figure 4, the near subsurface region (*∼*500 nm) of the PRR-1k1M-treated wire displays diaphite-like characteristics, whereas below this point the backbone wire structure now comprises regions that are purely sp^2^ (amorphous or ordered) within diamond without the diaphite interfacial region. A plausible explanation for the observed electrical characteristics would therefore be that this change in the nature of the NCNs created subsurface leads to a junction between metallic and semiconducting phases; this, in turn, leads to the diode-like behavior. That the diaphite phase does not behave this way when present in a continuous form (PRR-1k, Figure 2) can be explained by the overlapping, continuous, nondiaphite sp^2^ phases that are also present. Finally, it is worth reflecting on the stability of all three types of phase changes reported here. Previously reported work, using longer pulse picosecond laser radiation and without the aid of adaptive optics, has suggested from Raman spectroscopic measurements that the nanocarbon phases produced by that approach may be unstable with time.^48,49^ In our work, two lamellae were harvested from the same sample some six months apart, and the observations described here were entirely consistent between both; moreover, electrical measurements performed on the initial structures about one year later showed identical and stable characteristics. Of course, this is speculative at this stage but indicates an exciting scenario for the formation of 3D embedded active electronic structures within diamond. Indeed, this work indicates that the use of laser processing within the bulk of diamond substrates can be used for the formation of 3D written graphitic-like structures that can be made to display conducting, junction-like (semiconducting), or insulating electronic properties in a controllable and reproducible manner.

In terms of planetary science, it has been shown that conditions likely to be relevant to the lifecycle of meteorites and related space rocks can be recreated in the laboratory through the use of femtosecond laser radiation when it is highly confined within the body of a diamond matrix. To date, the observation of diaphite phases has been limited to micrometer-sized regions, at best, in, for example, meteorites. Here we have shown that extended diaphite phases can be produced, in the case of PRR-1K most likely throughout the near mm length of the laser-written wires. Indeed, it is possible that the use of the adaptive optics in the current study, whereby aberration correction results in a significantly more confined laser spot size within the diamond than conventional optical methods, may be a prerequisite for the observations made here, related to the formation of phases found in space-derived materials as well as offering the unique ability to vary the electrical properties of the 3D structures created.

## Conclusions

A particularly interesting feature of the current study is the observation of so-called “diaphite” carbon phases, both type 1 and type 2, that occur under differing experimental circumstances. “Type 1” is observed following the PRR-1k process that leads to an electrically conductive track. As discussed above, this type of diaphite structure has been associated with increasing the fracture resistance of diamond, which is typically low despite its compressive and tensile strength. While preventing the laser energy causing macroscale cracks and fractures, the fact that the type 1 diaphite also contains graphene-like carbon layers may explain why its presence is linked to the most conductive wires formed here. Under PRR-1M conditions (10^3^ increase is pulse delivery rate) the energy delivered to the system is simply too great for type 1 diaphite to mediate crack propagation, and a series of disconnected damaged regions form: surface cracks appearing to give way to the propagation of extensive networks of thread-like dislocations now accommodating the strain caused by the laser-induced phase transformations. The somewhat counterintuitive observation that this wire no longer electrically conducts despite receiving far greater laser energy for phase transformation can now be understood. The observation of regions that appear similar to the reported diaphite type 2 suggests that this phase forms under the distinctly different energy deposition conditions of the PRR-1M process compared to the PRR-1k process; further work on this aspect may offer useful insight into the origin of these complex nanocarbon phases found in space rocks and meteorites. The final condition explored was the overwrite of a PRR-1K wire with PRR-1M, a process referred to here as PRR-1k1M. Here, in the immediate subsurface region from the laser-write initiation side diaphite type 1 can be observed, but deeper into the overwritten laser structure no diaphite-like phases were seen. The junction-like electrical properties that resulted could be reproducibly created in several places on the sample and other samples. As such, it appears that the physical processes occurring immediately subsurface can be mediated by diaphite structures, but once the beam is deeper into the material, the diamond has insufficient relaxation through diaphites and forms sp^2^/sp^3^regions with sufficient connectivity to be conductive. In such a case the interface where such a switch-over occurs would be responsible for the junction-like electrical behavior. Significant further study is required to understand the various processes at work here, but in terms of technology, a stable 3D junction has been created within the body of a diamond. Since the ability to write conductive, semiconductive, and insulating wires in 3D within the body of a diamond substrate has been demonstrated, a possibly paradigm-changing all-carbon system for electronics and other forms of device can be contemplated. Further, fundamental issues relating to the appearance of diamond and related nanocarbons in space rocks have been opened through the utilization of this technological approach.

## Methods

### Substrate Preparation

Prior to laser processing, electronic grade single-crystal diamond samples (Chenguang Machinery & Electric Equipment Co. Ltd., China, 4 × 4 × 0.5 mm) were cleaned using the process described in Baral et al.^49^ Briefly, substrates were submerged in a saturated solution of sulfuric acid and ammonium peroxydisulfate at 200 °C for 20 min, followed by a rinse solution of hydrogen peroxide and ammonium hydroxide (1:1) for 10 min. This process works both to remove any surface contaminants and to etch any sp^2^ carbon species present on the substrate surface. To check for reproducibility, several tracks were drawn on the same substrate under the conditions described below, and subsequently, the measurements were repeated on other single-crystal diamond substrates following this type of laser processing. At all times, the observations were repeatable. Further, repeat measurements made about one year later on the original laser-processed material showed no change in *I*–*V* measurements, indicating the long-term stability of the electrical properties reported here.

### Laser Processing

The experimental system for laser processing of the NCNs is shown in Figure S3. The pulse energy from the ultrashort pulsed Yb:KGW laser (Light Conversion Pharos SP-06-1000-pp) (λ = 515 nm, 170 fs) was controlled using a rotatable half waveplate and polarizer. The beam was expanded and directed onto a phase-only liquid crystal spatial light modulator (SLM, Hamamatsu X10468), which was imaged in 4f configuration onto the back of a microscope objective lens (Zeiss 20×, 0.5 NA). In this manner, the laser could be focused down to a diffraction-limited spot size of 0.6 λ/NA = 0.6 μm in the transverse plane and 2*n*λ/NA^2^ = 9.8 μm axially (inside the diamond, where the refractive index *n* = 2.4).

The diamond sample was mounted on 3D precision translation stages (Aerotech ABL10100 (*x*, *y*); ANT95-3-V (*z*)). Columns of NCN were fabricated by focusing the laser on the backside of the diamond wafer and drawing the laser focus through to the top side at a sample translation speed of 10 μm/s. Note that this is the speed, *v*_s_, at which the sample physically moves, while the laser focus moves faster inside the diamond because of refraction at the interface. When focusing at NA = 0.5, the speed, *v*_a_, inside the diamond is well approximated by *n* × *v*_s_ = 24 μm/s.

The sample was illuminated from underneath by a red LED. A dichroic mirror separated the LED illumination from the laser before passing through a tube lens to a CCD, forming a microscope to allow imaging of the diamond sample during fabrication.

In general, during laser processing, strong spherical aberrations are introduced by refraction at the diamond interface which distort the laser focus and lead to reduced performance.^19,23^ The SLM was used for aberration correction, by predistorting the phase of the input laser beam to cancel the spherical aberrations, ensuring diffusion-limited performance throughout the diamond layer. We note that the SLM was only used to remove spherical aberration components of the interface aberration, while not compensating for the refocusing effect of the interface.^50^ After compensating for system aberrations by minimizing the fabrication threshold on the upper surface of the diamond sample, the aberration correction was done in a predictive manner using the position feedback of the axial translation stage.

### Electrical Characterization

Ti–Pt–Au (20:5:200 nm thickness) contacts were deposited (Edwards A500 - FL500 electron beam evaporator) and annealed at 400 °C for 1 h to form reliable ohmic contacts for probing. The contacts were patterned via a photolithographic lift-off process (Heidelberg DWL 66+). Current–voltage measurements were recorded using a Tektronix Keithley 4200-A SCS and an Evergreen EB-6 probe station supplied by Lambda Photometrics Ltd.

### TEM Imaging

Electron-transparent lamellae were made using a FIB by a lift-out method^34^ using a Scios 2 dual beam microscope from Thermo-Fisher. The lamellae were extracted from the center of each laser incident point from the rear (initiating) face, easily observable by SEM. The lamellae were 15 μm long by approximately 5 μm deep with a thickness of around 70 nm. The process is illustrated in the schematic form in Figure S4. Two lamellae were produced at a time period of ca. six months apart. In both cases the trends reported here were consistently observed in both, and, given the time between lamellae harvesting, the nanocarbon phases can be considered to be stable, at least over this time period and under room temperature and pressure conditions.

Electron microscope observations were carried out using two pieces of FEI equipment. Specifically, HRTEM and STEM studies were performed under a Talos F200X, and HRSTEM studies were carried out under a Cubed Titan Themis 60-300. Both electron microscopes are equipped with a high-brightness XFEG gun, and the last one also is equipped with a double correction system (Cs-DCOR) for probe and image. All of the electron microscopy experiments were run at 200 keV as the electron acceleration voltage. Velox software was used to process TEM micrographs.

## Supporting Information Available

Figures showing TEM images of a region lower down in the diamond FIB lamella in sample of setting PRR-1k, with the inset showing experimental laser setup; transmission and polarized optical microscope images of the top surface of the diamond substrate, showing the three types of laser-written columns; detailed technical information on the actual optically illumination arrangement used in the form of a schematic with all optical components identified; lift-off methodology for the preparation of TEM lamella in diamond materials in both schematic and SEM image forms (PDF)
