# Supplementary material for: Laser Engineering Nanocarbon Phases within Diamond for Science and Electronics
Source: ACS Nano. 2024 Jan 17;18(4):2861–71. doi: 10.1021/acsnano.3c07116 (PMC10832029; doi:10.1021/acsnano.3c07116)
Supplement: Supplementary file 1 — nn3c07116_si_001.pdf [file nn3c07116_si_001.pdf]

Supplementary information

Laser engineering nanocarbon phases within diamond for science and electronics

Patrick S. Salter[1†], M. Pilar Villar[2†], Fernando Lloret[2], Daniel F. Reyes[2], Marta Krueger[1], Calum S. Henderson[3], Daniel Araujo[2] and Richard B. Jackman[3*]

[1]Department of Engineering Science, University of Oxford, Parks Road, Oxford, OX1 3PJ, UK.
[2]Department of the Science of Materials, University of Cadiz, 11510, Puerto Real, Spain.
[3]London Centre for Nanotechnology and Department of Electronic and Electrical Engineering, UCL (University College London), 17 - 19 Gordon Street, London, WC1H 0AH, UK.

**Corresponding author. E-mail: r.jackman@ucl.ac.uk*
†These authors contributed equally to this work.

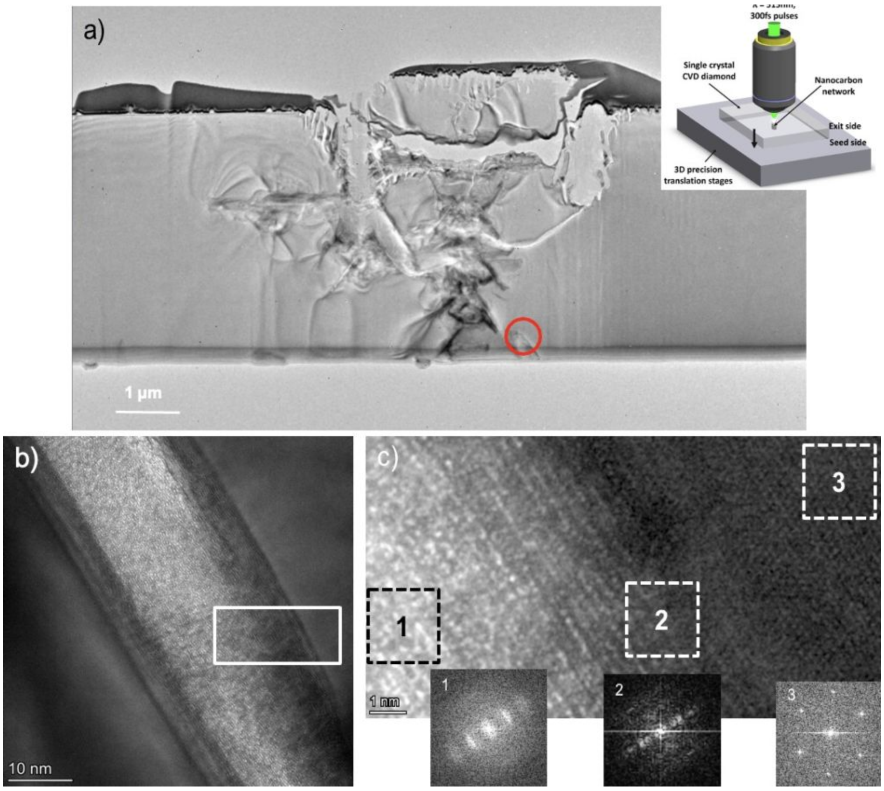

Fig. S1 (a) General TEM image of a region lower-down in the diamond FIB lamella in sample of setting PRR-1k, with inset showing experimental laser set-up. As referred to in the main text, the FIB lamella was considerably thicker in this region, reducing the resolution possible. However, as indicated in (b) and (c), structures similar to those in Figure 2 can be seen. (b) High-resolution micrograph of area surrounded by red circle in Fig. 1a. (c) Average filtered image of cropped and zoomed rectangle region in (b), with corresponding FFTs inside the graphitic channel (1), interphase channel-diamond (2) and single-crystal diamond (3).

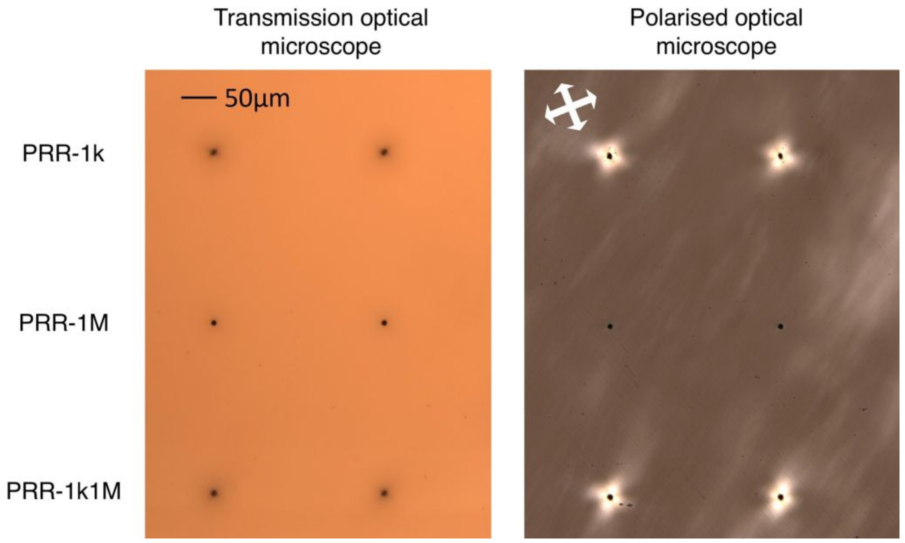

Fig. S2 Transmission (left) and polarized (right) optical microscope images of the top surface of the diamond substrate, showing the three types of laser-written columns. The bright portions surrounding the PRR-1k and PRR-1k1M NCNs is indicative of the strain placed upon the surrounding diamond crystal lattice due to the production of the lower density graphitic structures. The same strain is not present around the PRR-1M material, however, complimenting the conclusions drawn from the STEM images which showed a less graphitic nature when compared to the other laser settings.

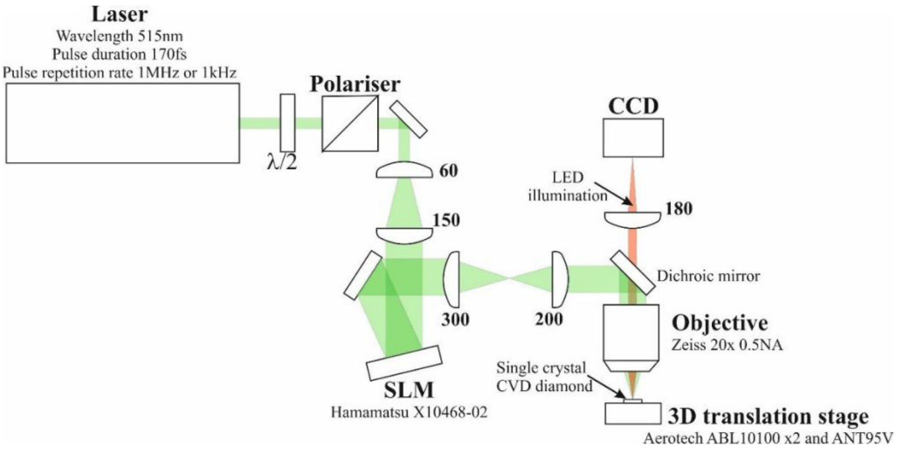

Fig. S3 The experimental system for laser processing of the NCNs. All lenses are achromatic doublets, with focal lengths as indicated next to each element.

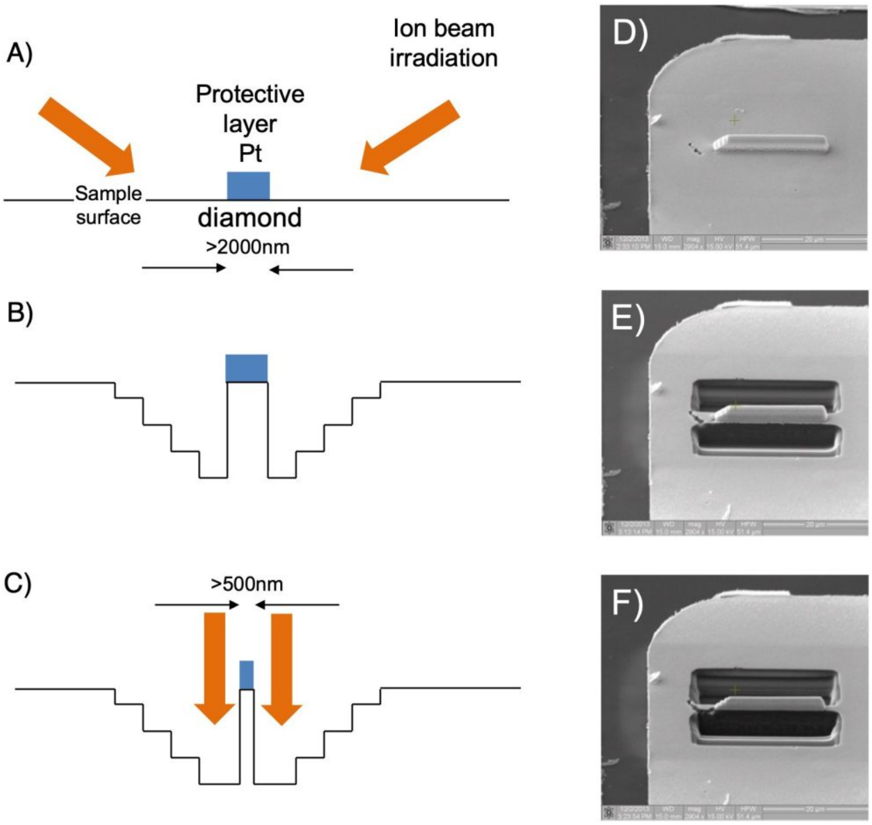

Fig. S4 Lift-off methodology for the preparation of TEM lamella in diamond materials. After Pt deposition for surface protection, two parallel trenches are milled at both sides, and, after cutting off and removing using a micromanipulator tip, final polishing is performed.
